# Endometrial factors and pregnancy loss frequency in recurrent pregnancy loss patients: comparing RT-PCR microbiology, microbial cultures, and immunohistochemistry of endometrium biopsy

**DOI:** 10.1007/s13353-025-00949-5

**Published:** 2025-02-20

**Authors:** K. Klimaszyk, P. Wirstlein, K. Bednarek-Rajewska, M. Jankowski, H. Svarre Nielsen, E. Wender Ożegowska, M. Kędzia

**Affiliations:** 1 Department of Reproduction, Chair of Reproduction and Perinatal Medicine, Poznan University of Medical Sciences, 61-701 Poznan, Poland; 2 Department of Clinical Pathology, Poznan University of Medical Sciences, Poznan, Poland; 3 Greater Poland Center of Digital Medicine, Poznan University of Medical Sciences, 61-701 Poznan, Poland; 4 The Recurrent Pregnancy Loss Unit, The Capital Region, The Fertility Clinic, Copenhagen University Hospitals Rigshospitalet and Hvidovre, Copenhagen, Denmark; 5 The Department of Clinical Medicine, University of Copenhagen, Copenhagen, Denmark; 6 Doctoral School, Poznan University of Medical Sciences, Poznań, Poland

**Keywords:** Endometrial microbiome, RT-PCR, Microbial culture, Immunohistochemistry, CD138, Chronic endometritis, Recurrent pregnancy losses

## Abstract

The objective of this study is to investigate the presence of bacteria in endometrial samples from patients with recurrent pregnancy loss (RPL) and explore potential correlations between bacterial presence, chronic endometritis, and previous pregnancy loss history. Endometrial samples from 90 RPL patients were analysed using RT-PCR to detect 10 specific bacterial species. A subgroup of 65 patients underwent additional microbial culture and immunohistochemistry for plasma cell identification. Correlations between bacterial presence, chronic endometritis, and the number of previous pregnancy losses were evaluated. We detected at least one out of 10 chosen bacteria DNA by RT-PCR in 24.4% (22/90) of endometrial samples. Patients with PCR-identified bacteria had a significantly higher number of previous pregnancy losses (median 3 vs 2, *p* = 0.01). No correlation was observed between bacterial presence and chronic endometritis diagnosis. A significant correlation was found between bacterial detection by PCR and microbial culture (*p* = 0.03), though culture methods detected fewer positive cases. In RPL patients, detecting DNA from at least one of 10 selected bacterial species by RT-PCR correlates with a higher number of previous pregnancy losses. However, this bacterial presence does not correlate with chronic endometritis diagnosis based on the CD138 immunohistochemistry-identified plasma cell count. These findings suggest a potential role of endometrial bacteria in RPL that may be independent of the classical inflammatory response associated with chronic endometritis.

## Introduction

Recurrent pregnancy loss (RPL), characterised by the loss of two or more pregnancies, is a growing area of research due to its significant impact on patients. RPL affects approximately 1 to 5% of women of reproductive age, with estimates suggesting that around 5% may experience two or more consecutive miscarriages, while only 0.4 to 1% have three or more losses. While specific prevalence rates for Poland are not universally reported, studies suggest that the incidence aligns with global figures, indicating that RPL is a notable concern among Polish women (Turesheva et al. 2023; Wojcicki et al. 2023). The complex interplay between the maternal host and the developing embryo highlights the crucial role of the endometrial microenvironment in early embryo-endometrial interactions (Salamonsen et al. 2016; Odendaal et al. 2024).

Recent studies have challenged the long-held belief that the uterine environment is sterile, revealing the presence of a microbiome that may play a crucial role in reproductive outcomes (Takimoto et al. 2023; Chen et al. 2017; Oshina et al. 2023). Advances in molecular technology have enabled the exploration of this endometrial microbiome, suggesting potential links to various gynaecological conditions and reproductive failures.

Previous research has associated bacteria identified in endometrium samples by microbial culture**,** particularly involving *Streptococcus* species, *Escherichia coli*, *Enterococcus faecalis* and *Staphylococcus* species, *Mycoplasma*/*Ureaplasma* species, *Proteus* species, *Pseudomonas aeruginosa*, *Klebsiella pneumoniae*, *Gardnerella vaginalis*, *Corynebacterium* species, and yeast with presence of chronic endometritis (CE) (Cicinelli et al. 2009; Kitaya et al. 2017). CE, characterised by migrating plasma cells and B lymphocytes to the endometrial stroma, has been linked to RPL, recurrent implantation failure, and infertility (Santoro et al. 2023).

The diagnostic criteria for CE lack international consensus, making research in this field challenging due to varying histopathological thresholds used across studies (Margulies et al. 2021; Klimaszyk et al. 2023; Huang et al. 2020).

Recent research has demonstrated a correlation between dysbiotic uterine microbiota and adverse pregnancy outcomes. These studies have primarily focused on alterations in the composition and diversity of endometrial microbiota, rather than on specific bacterial species. In particular, Lactobacilli composition has emerged as a crucial factor in creating a favourable endometrial environment for embryo implantation and growth (Takimoto et al. 2023; Moreno et al. 2016; Zeng et al. 2022).

Our study examines the presence of chosen pathogenic CE bacteria in endometrial biopsy samples from RPL patients using RT-PCR identification techniques and microbial culture methods. Additionally, we explore the immune response of the endometrium using immunohistochemistry staining for plasma cell detection for chronic endometritis diagnosis.

Our study encompasses 98 women of reproductive age with a history of two or more pregnancy losses before the 22nd gestational week. We conducted diagnostic hysteroscopies with subsequent endometrial biopsies during the follicular phase. The samples were analysed using PCR identification, microbial culture methods, and immunohistochemistry staining to identify plasma cells as a diagnostic marker for CE.

This research aims to provide insights into the prevalence and types of bacteria present in the endometrium of women with RPL, contributing to the growing body of evidence on the role of endometrial bacteria in reproductive health and giving more insight into aetiology of CE as an endometrial immunological response.

## Materials and methods

### Study population and sample collection

Participants in this study were 98 Caucasian women of reproductive age undergoing recurrent pregnancy loss (RPL) investigation at the Department of Reproduction in Gynecologic and Obstetrical University Hospital in Poznan, Poland, during the period from 2022 to 2024. Inclusion criteria were a history of two or more pregnancy losses before the 22nd week of gestation, reproductive age, and willingness to attempt subsequent pregnancy. In this study, we defined pregnancy loss as either biochemical pregnancies or clinically confirmed intrauterine pregnancies, excluding ectopic pregnancies. We studied two categories of recurrent pregnancy loss (RPL): primary RPL, defined as multiple pregnancy losses in patients who had never had a viable pregnancy, and secondary RPL, defined as multiple pregnancy losses in patients who had experienced at least one successful pregnancy. We performed diagnostic hysteroscopy, as a part of routine uterine cavity assessment with subsequent endometrial biopsy using a Pipelle de Cornier during the follicular phase of the menstrual cycle. To minimise contamination risk, a vaginal speculum was inserted, the vagina and external cervical os were cleaned with chlorhexidine-soaked gauze and the Pipella was inserted under visual control into the uterine cavity avoiding any contact with the vaginal walls. We obtained 90 good-quality endometrial samples for RT-PCR testing.

We conducted real-time polymerase chain reaction (RT-PCR) on all 90 endometrial samples to detect 10 specific bacterial species, replicating the panel used by Moreno et al. in their 2018 study.

**Fig. 1:**
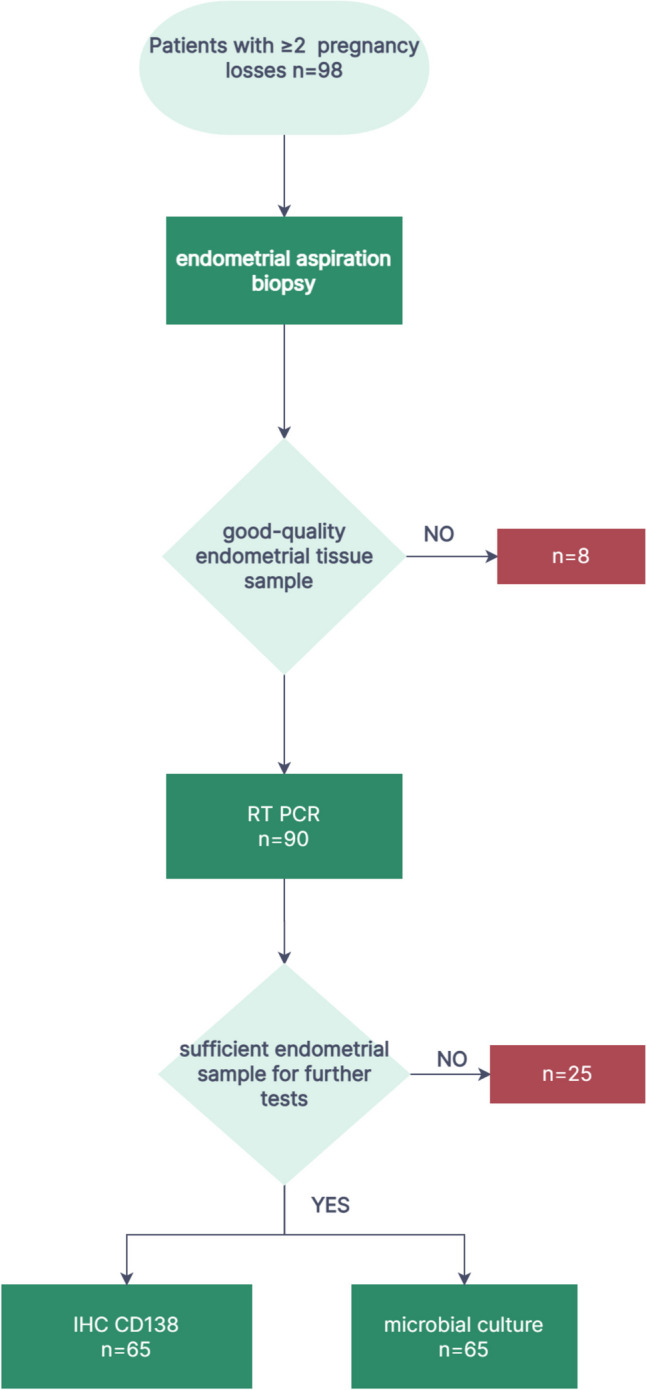
Flow chart of patient recruitment and performed examinations. RT-PCR, real-time polymerase chain reaction; IHC CD138, immunohistochemistry using CD138 antibody

Of the 90 endometrial samples suitable for RT-PCR testing, 65 were of sufficient quantity and quantity to allow for further analyses. These additional tests included microbial cultures and immunohistochemistry (IHC) staining using CD138 antibody for plasma cell identification (Fig. 1).

Samples were processed according to current standards for microbiological culture, using separate tests to detect both culturable and non-culturable bacterial strains.

### PCR identification techniques

#### DNA isolation, standard DNA, controls, and quantitative PCR (qPCR) method

For DNA isolation from the biopsy, the Bacterial & Yeast Genomic DNA Purification Kit (EURx, Poland) and the BeadTubeDry homogenization kit (EURx, Poland) were used. The procedure was performed according to the manufacturer’s instructions. The concentration of the obtained DNA isolate for each sample was measured using a spectrophotometer (NanoPhotometer N60, Germany) at a wavelength of *l* = 260 nm. The concentration of the extracted DNA ranged from 45 to 372 ng/uL. The isolates were stored at 2–8 °C until the PCR reaction was performed. Standard bacterial DNA for quantitative PCR and positive controls were obtained from the Leibniz Institute DSMZ-German Collection of Microorganisms and Cell Cultures GmbH, Germany. Human DNA was isolated from the peripheral blood of a healthy, anonymous male donor using the same protocol as for test samples. Quantitative PCR (qPCR) was employed to refine the identification of selected bacteria. Reactions were performed using SG qPCR Master Mix (2x) (EURx, Poland). Standard curves were prepared using serially diluted bacterial DNA (10, 1, 10^−1^, 10^−2^, and 10^−3^ ng/µL) in a solution of 100 ng/µL human DNA in TE buffer. A concentration of 0 ng/µL of pure 100 ng/µL human DNA solution in TE buffer served as the negative control (Table 1).

**Table 1:** The reaction mixture composition

Component	Volume (µL)	Final concentration
SG qPCR Master Mix (2x)	12.5	2.5 mM MgCl
Primer forward	1	0.5 µM
Primer reverse	1	0.5 µM
Template DNA	5	< 100 ng DNA
H2O	5.5	-
Total volume	25	-

qPCR cycling conditions were initial denaturation at 95°C for 5 min, followed by 40 cycles of denaturation (95°C for 15 s), annealing (60°C for 30 s), and extension (72°C for 30 s). Fluorescence data were collected during the annealing/extension step. The primer sequences for identifying the tested bacteria are presented in Table 2.

**Table 2:** Primer sequences and annealing temperatures. *rRNA* ribosomal RNA. The annealing temperature (Tm^0^C) for the primer pair was determined using an online calculator

Bacteria	Gene	Primer sequences	Amplicon size (bp)	Tm^0^C
*Chlamydia trachomatis*	16S rRNA	F: GGATCCGTAAGTTAGACGAAATTTTG R: TTTAATGCGAAAGGAAATCTGATTG	83	56
*Enterobacteriaceae*	*rpoB*	F: CAGGTCGTCACGGTAACAAG R: GTGGTTCAGTTTCAGCATGTAC	512	50
*Enterococcus* species	*rpoB*	F: AGAGAGTAAGGTCCGATTGAAC R: GGTTGTTTCCCGTATTATGC	370	53
*Escherichia coli*	16S rRNA	F: AGAAGCTTGCTCTTTGCTGA R: CTTTGGTCTTGCGACGTTAT	120	55
*Gardnerella vaginalis*	16S rRNA	F: TTACTGGTGTATCACTGTAAGG R: CCGTCACAGGCTGAACAGT	320	51
*Klebsiella pneumoniae*	*gltA*	F: ACGGCCGAATATGACGAATTC R: AGAGTGATCTGCTCATGAA	68	57
*Mycoplasma hominis*	16S rRNA	F: CATGCATGTCGAGCGAGGTT R: CCATGCGGTTCCATGCGT	129	55
*Neisseria gonorrhoeae*	16S rRNA	F: GTTTCAGCGGCAGCATTCA R: CCGGAACTGGTTTCATCTGATT	102	55
*Staphylococcus* species	*rpoB*	F: CAGGAGAAGTTAAAGAACAAGAAG R: GTGAACGAACTAATTGAGATACG	118	54
*Streptococcus* species	*tuf*	F: GTACAGTTGCTTCAGGACGTATC R: ACGTTCGATTTCATCACGTTG	197	54

Analysis of each sample included negative and positive controls and no template control to verify potential contamination and nonspecific amplification. Positive controls included a mixture of all DNA standards of the bacteria tested at a concentration equivalent to 10,000 genomes each and 100 ng/µL human genomic DNA. Negative controls included all DNA standards and 100 ng/µL human genomic DNA except for the microorganism standard evaluated in each assay. No template control contained H2O instead of DNA solution. After amplification, melting curve analysis was performed to distinguish target PCR products from nonspecific PCR products. The concentration of microorganism-specific DNA in each sample was determined by relating the Ct values in the qPCR cycle to the microorganism-specific standard curve, and the resulting concentration was then converted to genome equivalents (GE).

### Microbial culture

The material for microbiological culture was secured against contamination and delivered to the laboratory immediately after collection at room temperature. The sample was homogenised in a sterile homogeniser with the addition of 0.5 mL of sterile BHI broth. A preparation was made from the obtained homogenate, and Gram staining was applied. The homogenate was then plated onto various media: BHI broth, Columbia agar with sheep blood, *Gardnerella* agar, chocolate agar with polyvitex, Mycoplasma IST2, and Sabouraud agar with chloramphenicol and gentamicin. The incubation of the cultures was carried out under different temperature and atmospheric conditions, depending on the medium used. The identification of microorganisms included urogenital mycoplasmas, yeast-like fungi, staphylococci, enterococci, Gram-negative and Gram-positive rods, gonococci, and *Chlamydia trachomatis*, using tests and the VITEK2 analyser from bioMérieux.

### Immunohistochemistry

Endometrial samples were fixed in buffered 4% formaldehyde, dehydrated through an ethanol gradient, cleared in xylene, and embedded in paraffin. Three 4 µm sections from each sample were cut for immunohistochemical staining and analysis using SuperFrost®plus (Menzel Gläser) adhesive slides.

Immunohistochemistry staining used CD138 (EP201 clone) Rabbit Monoclonal Antibody (Cell Marque) following the Ready To Use Kit (RTU Kit) procedures. Tonsil tissue served as a positive control. Staining was performed with an ultraView DAB Detection Kit (Roche/Ventana) using the validated Roche protocol for the Benchmark Ultra slide staining system. The fixation time of the endometrial samples varies from 6 to 72 h depending on the size of the specimen. The fixation time of our samples was 24 h.

Plasma cells were identified by strong positive membrane staining with weak or no cytoplasmic staining. Plasma cells showed the presence of an ‘off-centred’, round nucleus with characteristic thick radially arranged chromatin forming a ‘bicycle wheel’ pattern. Chronic endometritis was diagnosed by at least one plasma cell per 10 high-power fields (HPF), with one HPF corresponding to 0.55 mm^2^.

### Statistical analysis

Statistical analyses were performed using PQ Stat® Statistical Software version 1.8.6. The normality of the distribution of the analysed parameters was assessed using the Shapiro–Wilk test. The Mann–Whitney *U*-test was used for the statistical analysis of numerical variables. Fisher’s exact test was used to estimate the association between binary variables.

A binary coding system was implemented for PCR-based bacterial identification: ‘1’ for detecting at least one out of 10 bacteria and ‘0’ for no bacteria detected.

The study investigated potential correlations between bacterial presence (as determined by PCR) and the number of plasma cells in endometrial sections, pregnancy losses, and positive microbial cultures in endometrial samples.

A *p*-value ≤ 0.05 was established as the threshold for statistical significance.

## Results

### Study population

Our study analysed 90 high-quality endometrial samples from patients diagnosed with recurrent pregnancy loss (RPL). The characteristic of the study group is presented in Table 3.

**Table 3:** Group characteristic

Number of examined endometrial samples	90
Age, years (mean; range)	32.8 (21–46)
BMI, kg/m^2^ (mean; range)	24.8(17.3–37.4)
Number of intrauterine pregnancy losses (median; range)	2 (2–6)
Gestational week of pregnancy loss, weeks (median; range)	7 (5–11)
Primary recurrent pregnancy loses	71% (64/90)
Secondary pregnancy losses	29% (26/90)

### RT-PCR bacterial detection

**Fig. 2:**
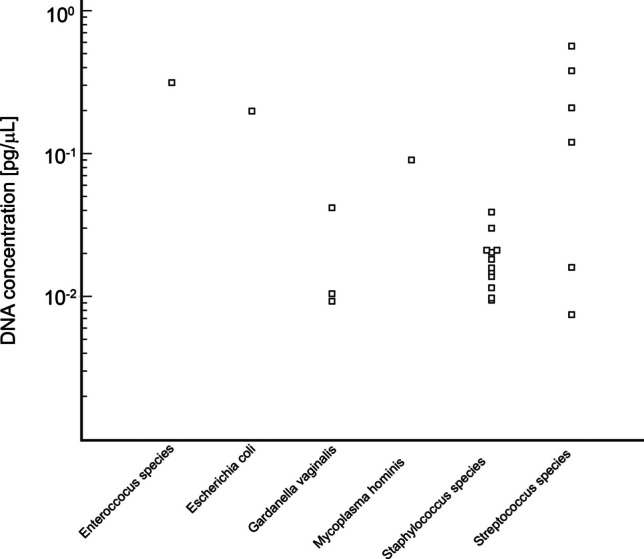
Bacterial DNA concentration measurements detected in endometrial samples

Using PCR identification methods, we detected at least one of 10 selected bacterial species by RT-PCR in 24.4% (22/90) of endometrial samples (Fig. 2). *Staphylococcus* species was the most prevalent bacteria, found in 11 samples, followed by *Streptococcus* species in 5 samples and *Gardnerella vaginalis* in 3 samples. *Enterococcus* species, *Escherichia coli*, and *Mycoplasma hominis* were each detected in one sample (Table 4).

**Table 4:** Number of endometrial samples with PCR-identified bacteria

Bacteria	Number of samples
*Staphylococcus* species	11
Streptococcus species	5
*Enterococcus* species	1
*Escherichia coli*	1
*Gardnerella vaginalis*	3
*Mycoplasma hominis*	1
*Neisseria gonorrhoeae*	0
*Klebsiella pneumoniae*	0
*Chlamydia trachomatis*	0

Our findings revealed that patients with PCR-identified bacteria exhibited a higher frequency of pregnancy losses (*p* = 0.01). The median number of pregnancy losses was 3 in the bacteria-positive group and 2 in the bacteria-negative group (Table 5).

**Table 5:** Demographic comparison of patients with and without PCR-identify bacteria

Clinical feature	PCR-identified bacteria	No PCR-identified bacteria	*p*-value
Number of samples	22	68	
Age, years (mean ± SD)	32 ± 4.54	33 ± 5.96	0.51
BMI, kg/m^2^ (median (IQR))	24 (21.4–29.8)	24 (21.2–26.6)	0.48
Number of intrauterine pregnancy losses (median (IQR))	3	2	**0.008**
Gestational week of pregnancy loss, weeks (median (IQR))	7.6 (6.56–9)	7.6 (6.17–8)	0.29
Primary recurrent pregnancy loses	15	45	0.77
Secondary pregnancy losses	7	23	

Bold values indicate statistically significant results (*p*<0.05)

### Comparison of PCR, immunohistochemistry, and microbial culture

**Fig. 3:**
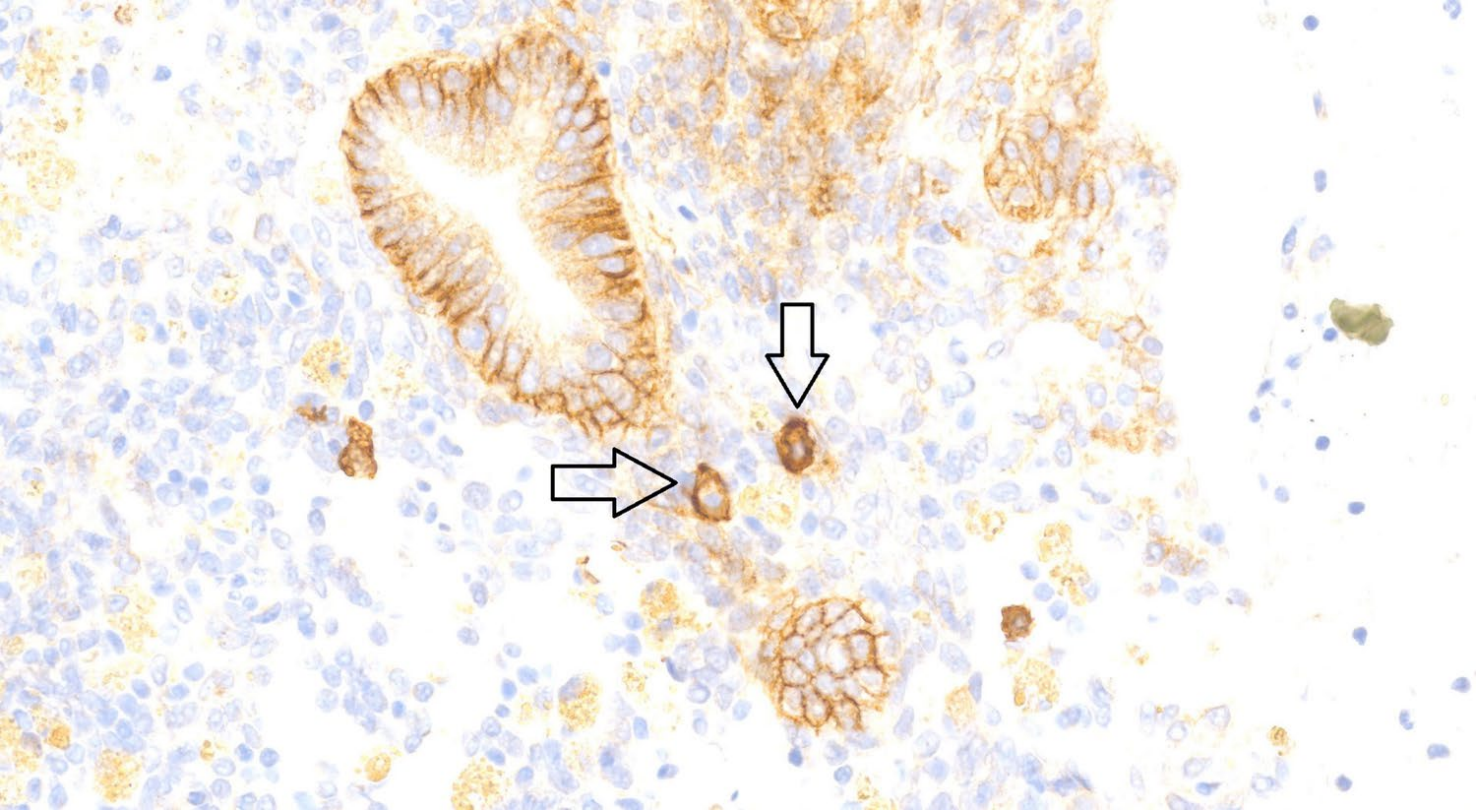
Immunohistochemical staining for CD138 in endometrial biopsy. Plasma cells show strong, membranous staining and weak cytoplasmic staining with CD138 antibody (arrows). Magnification × 400

In a subgroup of 65 patients, we performed PCR, microbial culture, and immunohistochemistry (IHC) for plasma cell identification. We assessed the morphological features of plasma cells stained with the CD138 antibody when the membrane showed strong positive staining while the cytoplasm showed weak or no staining (Fig. 3). No correlation was observed between bacterial presence in PCR examination and the number of plasma cells in endometrial sections. Given that chronic endometritis diagnosis is established by the presence of plasma cells in endometrial stroma, the observed lack of correlation between bacterial presence detected by PCR and plasma cell numbers (*p* = 0.27) consequently indicates no association between bacterial presence and chronic endometritis diagnosis. However, a significant correlation was found between bacterial detection by PCR and microbial culture (*p* = 0.03) (Table 6).

**Table 6:** Comparative analysis of endometrial samples from 65 recurrent pregnancy loss patients using PCR, immunohistochemistry, and microbial culture

Clinical feature	Bacteria presence detected using PCR	Bacteria presence not detected using PCR	*p*-value
Number of patients	18	47	n/a
Student’s *T*-test for independent samples			
CD138 (median (IQR))	0 (0–0.75)	0 (0–1.5)	0.27
Fisher’s exact test			
Microbial culture positive	5	3	**0.03**
Microbial culture negative	13	44	

Bold values indicate statistically significant results (*p*<0.05)

PCR-identified bacteria in 27.7% (18/65) of samples, while microbial culture was positive in 12.3% (8/65) of samples. Chronic endometritis, diagnosed based on IHC plasma cell identification, was present in 36.9% (24/65) of cases. In 28 patients, all three endometrial sample examinations were negative. Notably, only in three cases did RT-PCR identify the same type of bacteria as microbial culture (Table 7).

**Table 7:** Endometrial examination results for patients with one or more positive test outcomes

No	RT-PCR	Microbial culture	Plasma cells/10 HPF
1	-	*Staphylococcus hominis*	3
2	-	-	1
3	*Staphylococcus* species	-	1
4	*Gardnerella vaginalis*	-	0
5	*Staphylococcus* species	*Staphylococcus hominis*	1
6	*Streptococcus* species	*Streptococcus agalactiae*	2
7	-	-	3
8	*Mycoplasma hominis*	*Ureaplasma* spp./*Mycoplasma hominis*	0
9	-	-	5
10	-	-	1
11	-	-	4
12	*Staphylococcus* species	-	0
13	-	-	3
14	-	-	2
15	-	-	1
16	-	-	2
17	*Staphylococcus* species	-	0
18	*Staphylococcus* species	-	0
19	-	-	1
20	-	*Enterobacter cloacae* complex	2
21	*Staphylococcus* species	-	4
22	-	-	1
23	-	-	5
24	*Staphylococcus* species	-	0
25	*Escherichia coli* K12	*Escherichia coli*	0
26	*Staphylococcus* species	-	0
27	*Staphylococcus* species	-	0
28	*Gardnerella vaginalis*	*Lactobacillus gasseri*	0
29	*Staphylococcus* species	-	0
30	*Staphylococcus* species	-	1
31	-	-	1
32	-	*Candida kefyr*	3
33	-	-	2
34	-	-	1
35	-	-	2
36	*Streptococcus* species	-	0
37	*Streptococcus* species	-	0

## Discussion

Our study investigated the presence of bacteria in endometrial samples from patients with recurrent pregnancy loss (RPL) and explored potential correlations between bacterial presence and immunological response of the endometrium. Using RT-PCR identification techniques, we detected at least one of 10 selected bacterial species in 25.6% of endometrial samples. Notably, our results revealed a significant correlation between the presence of PCR-identified bacteria and a higher number of pregnancy losses, adding to the growing body of evidence that these bacteria may play a role in influencing reproductive outcomes.

In a subgroup of patients, we performed, in addition to PCR, two other types of endometrial biopsy examination: microbial culture and immunohistochemistry (IHC) for plasma cell identification. We observed no correlation between bacterial presence in PCR examination and the number of plasma cells in endometrial sections. However, a significant correlation was found between bacterial detection by PCR and microbial culture.

The role of endometrial factors in infertility and RPL has been the subject of extensive research, focusing on the immunological changes associated with chronic endometritis (Takimoto et al. 2023; Cicinelli et al. 2021; McQueen et al. 2015; Espinós et al. 2021). Studies have demonstrated associations between chronic endometritis and adverse pregnancy outcomes; however, the absence of standardised diagnostic criteria remains a contentious issue, potentially compromising the robustness of accrued evidence (Margulies et al. 2021; Klimaszyk et al. 2023; Huang et al. 2020).

Among several researchers addressing this diagnostic challenge, McQueen et al. conducted a study comparing CE prevalence between women with RPL and controls using expanded histopathological criteria, which notably included endometrial stromal changes alongside plasma cell assessment (McQueen et al. 2021). Their approach yielded optimal diagnostic accuracy when CE was defined by the presence of at least one plasma cell per 10 HPFs combined with stromal changes. A recent survey of pathologists’ clinical practices further highlights the global inconsistency in CE diagnostic criteria, underscoring the urgent need for standardisation, particularly as histopathological examination serves as a verification method for emerging diagnostic techniques (Margulies et al. 2021).

The aetiology of CE and its underlying immunological mechanisms remain incompletely understood. The initial concept of pathogenic bacteria as causative agents in CE development emerged from two key lines of evidence. First, the pioneering work of Cicinelli’s group identified bacteria in endometrial samples from CE patients using traditional microbial culture techniques (Cicinelli et al. 2009). Subsequently, Song et al. provided additional support for the infectious aetiology through a prospective randomised control trial demonstrating that antibiotic treatment effectively resolves histopathologically confirmed CE (Song et al. 2021).

Early microbial culture studies by Cicinelli and Kitaya’s research groups identified several common pathogens in CE patients, including *Streptococcus* species, *Escherichia coli*, *Enterococcus faecalis*, *Staphylococcus* species, *Mycoplasma*/*Ureaplasma* species, *Proteus* species, *Pseudomonas aeruginosa*, *Klebsiella pneumoniae*, *Gardnerella vaginalis*, *Corynebacterium* species, and yeast (Cicinelli et al. 2009; Kitaya et al. 2017; Cicinelli et al. 2008).

However, our understanding of endometrial microbiology has evolved significantly. When these initial studies were conducted, the uterine cavity was widely considered a sterile environment, leading researchers to conclude that any bacterial presence must be pathogenic and responsible for the inflammatory response seen in CE.

Our study design was inspired by the work of Moreno et al., who compared various diagnostic methods for chronic endometritis and introduced RT-PCR as a molecular detection technique in the CE diagnostic process (Moreno et al. 2018). Interestingly, Moreno et al. reported a higher rate of bacterial detection (55.79%) compared to our study (24.4%). This discrepancy in bacterial detection rates could be attributed to significant differences in the study populations. Our study focused exclusively on RPL patients. In contrast, Moreno et al. investigated infertile patients undergoing in vitro fertilisation (IVF) procedures. The bacterial profiles identified in our study and Moreno’s research reveal similarities and differences. Streptococci emerged as a common finding in both investigations. Moreno’s study identified Streptococci as the predominant pathogens, while our research found *Streptococcus* species to be the second most prevalent, present in 5 out of 22 positive samples. Notably, our study identified *Staphylococcus* species as the most frequently detected bacteria, found in 11 samples. Our investigation found isolated instances of *Enterococcus* species, *Escherichia coli*, and *Mycoplasma hominis*. Moreno’s study, however, did not provide a detailed breakdown of less prevalent pathogens, making a direct comparison challenging. Interestingly, neither study detected *Chlamydia trachomatis* or *Neisseria gonorrhoeae*.

Interestingly, we did not observe a correlation between the presence of bacteria detected by RT-PCR and chronic endometritis (CE) as defined by at least one plasma cell per 10 high-power fields (HPF) of the endometrial sample. This finding contrasts with a previous study by Liu et al. that compared the microbiota of CE and non-CE patients (Liu et al. 2019). However, it is essential to note that those studies employed different microbial analysis techniques, specifically culture-independent massively parallel sequencing of the 16S ribosomal RNA gene. Additionally, they used a different cut-off point for CE definition, which was > 5.15 cells per 10 mm^2^. Our study investigates the role of pathogenic bacteria and cannot be extrapolated to information on the uterus microbiome, that is most likely why we did not obtain similar results as Liu’s group.

Results from classical microbial culture techniques demonstrated a significant correlation with PCR findings. However, culture methods detected bacterial presence in fewer cases than RT-PCR. This discrepancy likely stems from the low abundance of bacteria in endometrial samples, which often falls below the threshold required for successful culture-based detection. This makes microbial culture a possible but unreliable method for detecting bacteria in endometrial samples.

It is essential to acknowledge that while PCR identification of specific bacteria provides valuable insights, it may not fully capture the complex relationship between the endometrial microbiome, chronic endometritis, and RPL. Although sensitive for detecting specific bacterial species, PCR-based methods have limitations in representing the full diversity and interactions within the microbiome.

Recent research has emphasised the importance of considering the entire microbial community rather than focusing on individual pathogens. For instance, Moreno et al. demonstrated that the overall composition of the endometrial microbiota, particularly the dominance of *Lactobacillus* species, could influence implantation success in IVF patients (Moreno et al. 2016). Chen et al. similarly found associations between altered endometrial microbiota and adverse reproductive outcomes (Chen et al. 2022).

These findings underscore the need for more comprehensive microbiome studies to elucidate the relationship between endometrial bacteria and RPL fully. Next-generation sequencing techniques, such as 16S rRNA gene sequencing, offer a more holistic approach to characterising the endometrial microbiome (Peuranpää et al. 2022). These methods can provide a more complete picture of microbial diversity and abundance, potentially revealing essential patterns or imbalances that may be missed by targeted PCR or culture-based techniques.

A compelling example of the importance of overall microbial community structure comes from a study by Tine Wronding et al., which demonstrated that vaginal microbiota transplantation, shifting from a *Gardnerella*-dominated to a *Lactobacillus*-dominant microbiome, resulted in successful pregnancies in RPL patient (Wrønding et al. 2023). This highlights the potential significance of the overall microbial community structure rather than the presence or absence of specific bacterial species. The case report presented by Moreno gives us the first glimpse into the microbiome of early pregnancy (Moreno et al. 2020). It shows that microbial profiles of endometrium differ in cases before miscarriage and before a successful pregnancy. *Lactobacillus iners* was the most prevalent microbe found in the endometrium during the fourth week of a successful pregnancy.

When studying endometrial microbiota using PCR, contamination poses significant challenges. This includes environmental contamination from high-biomass sites like the cervix and vagina, reagent contamination from extraction kits, and cross-contamination during sample handling. Given the low-biomass nature of endometrial samples, even minor contamination can significantly alter results (Reschini et al. 2022; Winters et al. 2019).

In our study, we conducted diagnostic hysteroscopy prior to endometrial sampling, following a similar approach used by several other research groups (Cicinelli et al. 2008; Moreno et al. 2018). Although a sterile medium was used during the hysteroscopy procedures, bacterial transfer through the cervical canal could still occur. However, since our microbial analysis was performed on endometrial tissue samples rather than uterine fluid, and we adhered to strict sterile protocols, we believe that any potential impact on our results was minimal.

While our study’s finding of a correlation between PCR-identified bacteria and increased frequency of pregnancy losses provides a valuable point, the future of research is more comprehensive microbiome analysis techniques. This approach could lead to a deeper understanding of the role of the endometrial microbiome in recurrent pregnancy loss and potentially inform new diagnostic and therapeutic strategies for women experiencing RPL.

## Limitations of the study

This study has several important methodological limitations. Our PCR analysis targeted only specific bacterial species described to be pathogenic for CE, which limits our ability to extrapolate findings regarding dysbiosis’s impact on endometrial immunological response and its correlation with RPL.

A significant technical challenge arose from the limited quantity of endometrial tissue obtained during biopsies in the follicular phase, when the endometrium is physiologically thinner. This prevented us from obtaining conclusive results from all planned analyses (PCR, immunohistochemistry, and microbial culture) in some cases.

Despite implementing strict sampling protocols with sterile speculums and chlorhexidine cleaning, the risk of contamination from vaginal flora and cervical canal during hysteroscopy remains an inherent limitation. Our sample size was determined pragmatically based on eligible patient recruitment during the study period, particularly affecting the interpretation of negative findings. Additionally, the lack of international consensus on chronic endometritis diagnostic criteria presents a broader challenge for comparing findings across studies and highlights the need for standardised approaches.

## Data Availability

The datasets analyzed during the current study are available from the corresponding author upon reasonable request.
